# 508. Nirmatrelvir/ritonavir use among patients with cancer and COVID-19 is associated with improved clinical outcomes: Single-institution case-control study

**DOI:** 10.1093/ofid/ofad500.577

**Published:** 2023-11-27

**Authors:** Panos Arvanitis, Kendra Vieira, Jeremy L Warner, Dimitrios Farmakiotis

**Affiliations:** The Warren Alpert Medical School of Brown University, Providence, Rhode Island; Divisions of Infectious Diseases, the Warren Alpert Medical School of Brown University, providence, Rhode Island; The Warren Alpert Medical School of Brown University, Providence, Rhode Island; Division of Infectious Diseases, The Warren Alpert Medical School of Brown University, providence, Rhode Island

## Abstract

**Background:**

Patients with cancer are at increased risk for severe and lethal COVID-19, compared to the general population. Currently, with no anti-spike monoclonal antibodies available against circulating variants of SARS-CoV-2, the logistic limitations of outpatient remdesivir infusions, and lack of efficacy of molnupiravir in vaccinated patients, nirmatrelvir/ritonavir (Paxlovid™) is the only effective oral therapy for outpatient use against COVID-19. However, its efficacy specifically in immunocompromised patients, including patients with cancer, has not been adequately studied.

**Methods:**

In this pilot analysis, we retrospectively studied the records of patients with history of or active cancer at Brown University-affiliated hospitals diagnosed with SARS-CoV-2 after Emergency Use Authorization (EUA, 2021-12-22) of Paxlovid™, and until 2022-07-21. Patients not meeting EUA criteria or receiving other outpatient antivirals were excluded (Figure 1). The primary outcome was 90-day COVID-19-attributed mortality, with 90-day all-cause mortality and hospitalization as secondary outcomes. Patients who died within 90 days from other causes were excluded from COVID-19-attributed mortality analyses.

Figure 1.

**Figure:**
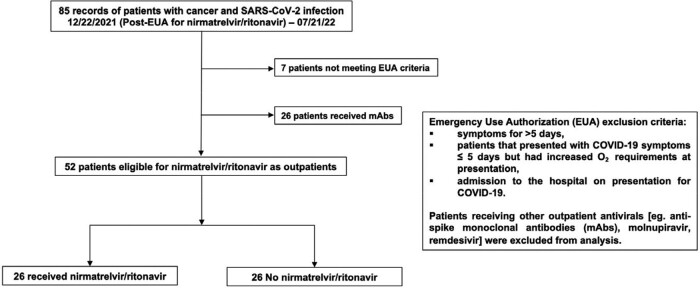

Flow diagram illustrating patient selection.

**Results:**

33 of 85 eligible patients were excluded (Figure 1). Of the 52 remaining, 26 received Paxlovid™. Baseline demographic and clinical characteristics were well-balanced between the two groups (Table 1). 50% were male, one was Black, five were Hispanic (10%); median age was 68 (interquartile range [IQR] 56-77) years; most patients (61.5%) had received 3 or more vaccine doses prior to infection. Paxlovid™ use was associated with numerically lower all-cause mortality rate (8% vs. 23%), and significantly lower rates of 90-day COVID-19-attributed mortality (0% vs. 19%, p=0.03; Log-rank p< 0.01, Figure 2), and hospitalization (27% vs. 65%, p< 0.01).

Table 1.

**Figure:**
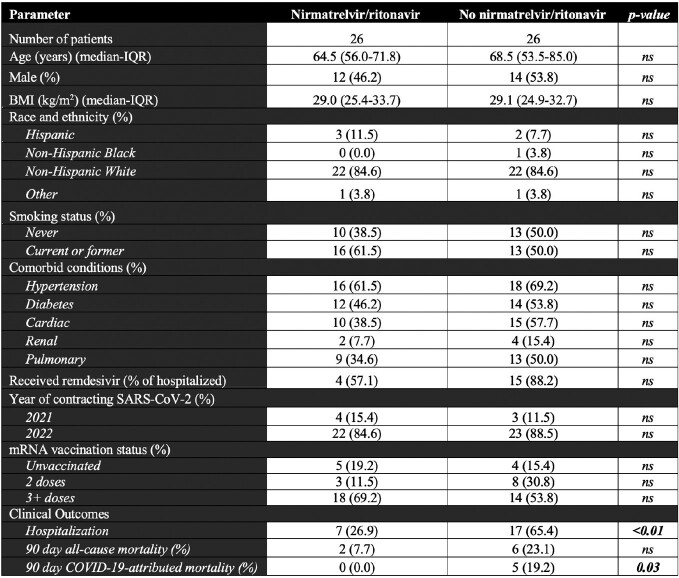

Baseline characteristics and clinical outcomes.

Figure 2.

**Figure:**
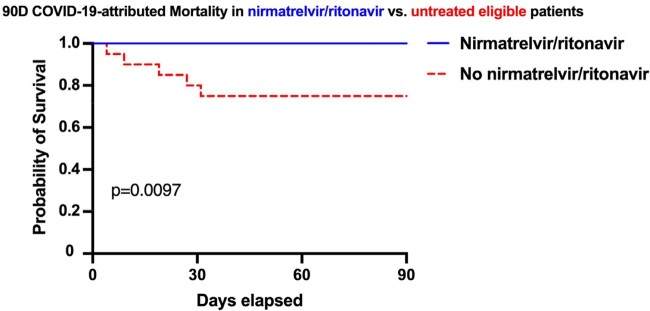

Kaplan-Meier survival curves.

**Conclusion:**

Paxlovid™ use was associated with a strong signal for improved clinical outcomes among patients with cancer and COVID-19, compared to similar contemporary controls, most of whom were fully vaccinated. Larger studies of Paxlovid™ use and efficacy in high-risk patients with cancer and other immunocompromised individuals are needed and ongoing.

**Disclosures:**

**Panos Arvanitis, MS**, NIH: Grant/Research Support|NIH: Brown University Summer Assistantship program and from the Brown Emerging Infectious Disease Scholars (EIDS) (5R25AI140490) **Jeremy L. Warner, MD, MS**, AACR: Grant/Research Support|Flatiron Health: Grant/Research Support|Melax Tech: Advisor/Consultant|NIH: Grant/Research Support|Roche: Advisor/Consultant|Westat: Advisor/Consultant **Dimitrios Farmakiotis, M.D.**, Astellas: Grant/Research Support|Merck: Grant/Research Support|Viracor: Advisor/Consultant|Viracor: Grant/Research Support

